# Enhanced Specificity in Colorimetric LAMP Assay for *Sarocladium kiliense* Detection Using a Combination of Two Additives

**DOI:** 10.3390/jof10120857

**Published:** 2024-12-11

**Authors:** Yu-Ning Wong, Pak-Ting Hau, Eddie Chung-Ting Chau, Laam-Ching Ng, Michaela Murillo, Joshua Fung, Wai-Wai Po, Ray Chun-Wai Yu, Melody Kai-Ting Kam, Emily Wan-Ting Tam, Chi-Ching Tsang, Franklin Wang-Ngai Chow

**Affiliations:** 1 Department of Health Technology and Informatics, The Hong Kong Polytechnic University, Hunghom, Hong Kong, China; yu-ning.wong@connect.polyu.hk (Y.-N.W.); pak-ting-patrick.hau@connect.polyu.hk (P.-T.H.); chungtingeddie.chau@connect.polyu.hk (E.C.-T.C.); laamching.ng@connect.polyu.hk (L.-C.N.); michaela.murillo@polyu.edu.hk (M.M.); wai-wai-christy.po@connect.polyu.hk (W.-W.P.); chun-wai-ray.yu@connect.polyu.hk (R.C.-W.Y.); 2 School of Applied Science, Nanyang Polytechnic, 180 Ang Mo Kio Avenue 8, Ang Mo Kio, Singapore 569830, Singapore; 3 School of Science and Technology, Hong Kong Metropolitan University, Homantin, Hong Kong, China; ewttam@hkmu.edu.hk; 4 School of Medical and Health Sciences, Tung Wah College, Homantin, Hong Kong, China

**Keywords:** LAMP, colorimetric, *Sarocladium kiliense*, molecular detection, combined additives

## Abstract

The genus *Sarocladium* comprises fungal species closely related to *Acremonium*, with *S. kiliense* and *S. strictum* being medically important. These species can cause infections in both immunocompetent and immunocompromised individuals. The current detection methods are limited, prompting the need for rapid and specific diagnostic tools. We developed a colorimetric loop-mediated isothermal amplification (LAMP) assay targeting *S. kiliense* (SK-LAMP). The initial prototype assay faced challenges with cross-reactivities with closely related species. To address this, we incorporated two additives, pullulan and tetramethylammonium chloride (TMAC), which are known to reduce non-specific signals in amplification assays. Our study found that a combination of 1% (*v*/*v*) pullulan and 0.03 M TMAC enhanced the specific detection of *S. kiliense* in a 45 min reaction, without non-specific false-positive results for other fungal species. This optimised LAMP assay demonstrated high sensitivity and specificity, offering a reliable and rapid method for detecting *S. kiliense*. The novel approach of combining additives to enhance assay specificity presents a promising strategy for improving LAMP assays targeting closely related fungal species. This advancement can aid in the timely diagnosis and management of infections caused by *S. kiliense*, contributing to better patient outcomes and infection control.

## 1. Introduction

The genus *Sarocladium* currently encompasses around 30 species, which are morphologically and genetically closely related to *Acremonium* [1,2,3,4]. While *Sarocladium* species are generally saprophytic fungi, which can be commonly found in the environment, several *Sarocladium* species are important pathogens in either plants or animals. For example, *S. attenuatum*, *S. oryzae* and *S. sinense* can cause diseases in rice (*Oryza sativa*) [5]. On the other hand, *S. kiliense* and *S. strictum*, which are capable of growing at 35–37 °C [6], are pathogens to both immunocompetent and immunocompromised individuals. These two medically important *Sarocladium* species have been isolated from various environmental sources, including drinking water, water taps [7,8], water storage tanks [9] and soil of recreational areas [10], posing a potential threat to the community. Localised diseases due to *Sarocladium* species, such as mycetoma, keratomycosis and onychomycosis, can be seen in immunocompetent patients, whereas invasive infections, such as fungaemia, peritonitis, pneumonia, endocarditis and central nervous system infection are reported in immunocompromised patients, often associated with high fatality [11,12]. Disseminated infections are also observed [11].

**Figure 1:**
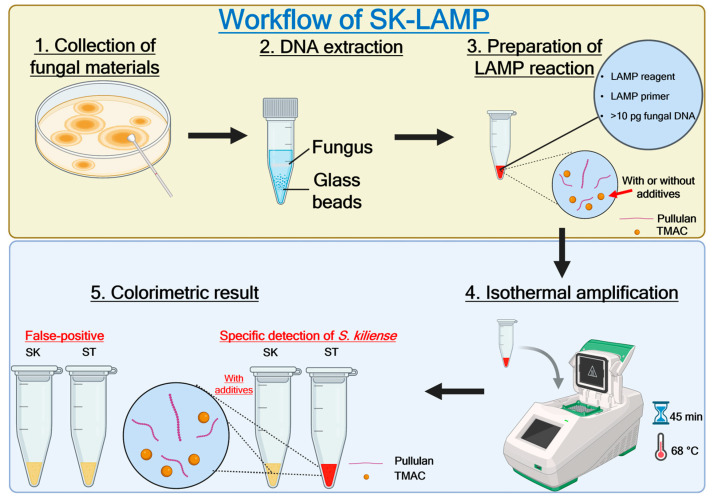
Schematic of the *Sarocladium kiliense*-specific loop-mediated isothermal amplification assay (SK-LAMP) workflow. Created in BioRender [42].

Despite their clinical significance, the true prevalence of *Sarocladium* infection is difficult to estimate due to incomplete species identification and/or inaccurate morphological identification methods. A previous study has highlighted the rarity of *Sarocladium* infection, where only 15 clinical isolates of *S. kiliense* were recovered from a reference laboratory in the US over an eight-year period [13]. However, as the morphology of *Sarocladium* can hardly be distinguished from *Acremonium* species [14], there may be an underestimation of the prevalence of *Sarocladium* infection, as cases may have been reported as *Acremonium* infection instead if only morphological but not molecular identification was performed. The most recent outbreak of *Sarocladium* was a nosocomial outbreak due to *S. kiliense*, which took place in multiple hospitals in Chile and Colombia during 2013–2014, involving more than 50 patients under chemotherapy who developed fungaemia following the intake of *S. kiliense*-contaminated antinausea drug [15]. While *Sarocladium* can be clinically important, no detection assay targeting *S. kiliense* has been developed to date. Currently, there is only one colorimetric loop-mediated isothermal amplification (LAMP) detection assay specific to the plant pathogen *S. oryzae* [16,17]. Therefore, the development of a rapid and reliable diagnostic tool is essential for the efficient management of *S. kiliense* infections. LAMP is a simple, fast and sensitive nucleic acid amplification technique that can be performed under isothermal conditions. While other isothermal amplification assays, such as rolling circle amplification (RCA) [18,19], recombinase polymerase amplification (RPA) [20,21,22,23], strand displacement amplification (SDA) [24,25], helicase-dependent amplification (HDA) [26,27,28] and nucleic acid sequence-based amplification (NASBA) [29,30], have also been developed for directly detecting nucleic acids of pathogens from host specimens, LAMP does not require expensive instruments, making it an affordable assay. LAMP is also more widely used and has been recommended for clinical use by the World Health Organization (WHO) for detecting certain pathogens, such as *Mycobacterium tuberculosis* and SARS-CoV-2 [17,31,32,33,34,35,36]. In this study, we established a colorimetric LAMP detection assay for *S. kiliense* and report the first use of two additives, pullulan and tetramethylammonium chloride (TMAC), in a LAMP reaction to enhance assay specificity (Figure 1). Though pullulan or TMAC have been widely used to rescue low sensitivity or specificity in nucleic acid amplification [37,38,39,40,41], there are no reports about the effects of combining both chemicals in one single reaction on nucleic acid amplification through the LAMP assay. This study provides a novel diagnostic tool for the rapid and reliable detection of *S. kiliense*, which may aid in the diagnosis and management of *S. kiliense*-associated infections.

## 2. Materials and Methods

### 2.1. Fungal Strains Used

Reference fungal strains *S. kiliense* (CBS 122.29^T^), *S. strictum* (CBS 346.70^T^),* S. summerbellii* (CBS 430.70^T^), *Acremonium egyptiacum* (CBS 114785^T^), *Aspergillus fumigatus* (CBS 101355), *Candida albicans* (CBS 8837), *Candidozyma auris* (CBS 10913^T^), *Fusarium keratoplasticum* (NRRL 43458), *Rhizopus microsporus* var. *chinensis* (CBS 344.29), *Talaromyces marneffei* (CBS 334.59^T^) and *Trichophyton mentagrophytes* (CBS 642.73) were purchased from the Westerdijk Institute (CBS; Utrecht, The Netherlands) or Agricultural Research Service (ARS) Culture Collection (NRRL; Peoria, IL, USA). They were cultured on Sabouraud dextrose agar (SDA; Oxoid, Hampshire, UK, Catalogue # CM0041) and/or potato dextrose agar (PDA; Oxoid, Catalogue # CM0139), both supplemented with chloramphenicol (50 μg/mL) (Sigma-Aldrich, Darmstadt, Germany, Catalogue # C0378), for 2–15 days at 25 ± 1 °C to obtain sufficient growth.

### 2.2. Fungal DNA Extraction

DNA extraction was performed as described previously [43]. Briefly, for each fungal isolate, approximately 300 mg of glass beads and 1 mL of TE buffer were added into a 2 mL screw-cap tube containing the cells. Fungal cells in the screw-cap tube were then disrupted by the Precellys Evolution tissue homogeniser (Bertin, Montigny-le-Bretonneux, France) at 8000 rpm for 10 s with a 5 s pause per cycle for six cycles. The homogenate was centrifuged at 1200× *g* for 5 min to obtain the supernatant, which contained microbial DNA. All the extracted DNA products were stored at −20 °C.

### 2.3. Primer Design

The internal transcribed spacer (ITS) region sequences of *Sarocladium* species type materials were retrieved from the RefSeq database and aligned to determine suitable DNA segments for primer design [44,45]. Multiple alignments of the ITS sequences were conducted through MEGA 11 [46]. LAMP primers were designed using Primer Explorer V5 from Eiken Chemical (Tokyo, Japan), with consideration regarding the optimal working temperature of *Bst* 2.0 WarmStart DNA polymerase that ranges from 60 to 70 °C. The primer sequences are provided as follows: F3 primer: 5′-GGGGACAACCAAACTCTGAT-3′, B3 primer: 5′-CCGAAAGGGGGTCCTGAG-3′, FIP primer: 5′-GCCAGAGCCAAGAGATCCGTTGTGAATCTCTGAGGGGCGA-3′ and BIP primer: 5′-TGAAGAACGCAGCGAAATGCGAGCGCAATGTGCGTTCAAAG-3′. All primers were synthesised by Invitrogen (Waltham, MA, USA).

### 2.4. Colorimetric LAMP

The initial prototype colorimetric LAMP assay was developed, without any additives, utilising the WarmStart^®^ Colorimetric LAMP 2× Master Mix (DNA & RNA) (New England BioLabs, Ipswich, MA, USA, Catalogue # M1800). The 10 μL colorimetric LAMP reaction consisting of 5 μL of WarmStart^®^ Colorimetric LAMP 2× Master Mix (DNA & RNA), 1 μL of 10× primer mixture [outer primer (F3, B3: 2 µM), inner primer (FIP, BIP: 8 µM)] (Invitrogen), 1 μL of DNA template and 3 μL of nuclease-free water was then incubated in the S1000 Thermal Cycler (Bio-Rad Laboratories, Hercules, CA, USA) at 68 °C for 45 min. A colour change from pink to yellow or amber was interpreted as positive, while the maintenance of a pink or coral pink colour was regarded as negative. The positive samples were confirmed by three additional technical replicates. Photographs were taken with the digital single-lens reflex (DSLR) camera Canon 650D equipped with the EF50 mm f/1.8 II lens on a light-emitting diode (LED) panel with a white background. The RAW CR2 files were processed in the Adobe Camera RAW software version 17.0.1, where the white balance and exposure were corrected globally across all photographs of the set. The white balance was set to 4650K, and a slight under-exposure was corrected after processing to preserve details in the shadows. No adjustments to hue and colour saturation were performed. The processed images were exported as JPG files. The CR2 and XMP files are available upon request.

### 2.5. Optimisation of the S. kiliense Detection Colorimetric LAMP Assay Using Single or Combined Additives

To improve assay specificity, additions of pullulan (1–2.5%, *v*/*v*) and TMAC (0.01–0.04 M) were tested and compared with the initial prototype assay lacking additives. DNA amounts of 1 ng, 0.5 ng and 0.1 ng from *S. kiliense*, *S. strictum* and *S. summerbellii* were included in the tests. The addition of both pullulan and TMAC was also evaluated.

The optimal combination was determined to be 1% pullulan and 0.03 M TMAC, and this was used for further evaluation of assay specificity and sensitivity. The reaction consisted of 10 μL of WarmStart^®^ Colorimetric LAMP 2× Master Mix, 2 μL of 10× primer mixture, 3.5 μL of water, 2 μL of 10% pullulan, 1.5 μL of 0.4 M TMAC and 1 μL of DNA template. LAMP reaction was performed at 68 °C for 45 min, followed by enzyme inactivation at 85 °C for 20 min. The analytical specificity was tested using 1 ng of DNA from eleven different fungal species, including *S. strictum, S. summerbellii*, *A. egyptiacum*, *A. fumigatus*, *C. albicans*, *C. auris*, *F. keratoplasticum*, *R. microsporus var. chinensis*, *T. marneffei* and *T. mentagrophytes*, whereas the analytical sensitivity was evaluated using 100 fg–10 ng of *S. kiliense* DNA.

## 3. Results

### 3.1. Detection of S. kiliense by Initial Prototype Colorimetric LAMP Assay

The initial prototype assay without any additive, performed under an incubation temperature of 68 °C for 45 min, successfully detected *S. kiliense* DNA at concentrations of 0.1–1 ng. However, it also detected *S. summerbellii* DNA at 1 ng and *S. strictum* DNA at <1 ng (Figure S1). Such detections of *S. strictum* and *S. summerbellii* DNA as false-positives revealed that this initial prototype colorimetric LAMP assay lacked the necessary specificity for *S. kiliense*. This prompted further evaluation of specificity-enhancing additives to improve the assay’s accuracy and ensure reliable differentiation between *S. kiliense* and other closely related *Sarocladium* species, especially *S. strictum*, which may also be of clinical relevance.

### 3.2. Effects of Additive Treatment for S. kiliense-Specific LAMP Assay (SK-LAMP)

While the initial prototype assay demonstrated the ability to detect *S. kiliense* DNA, it also amplified *S. strictum* DNA. To improve this, we evaluated the effect of specificity-enhancing additives in the LAMP assay.

**Figure 2:**
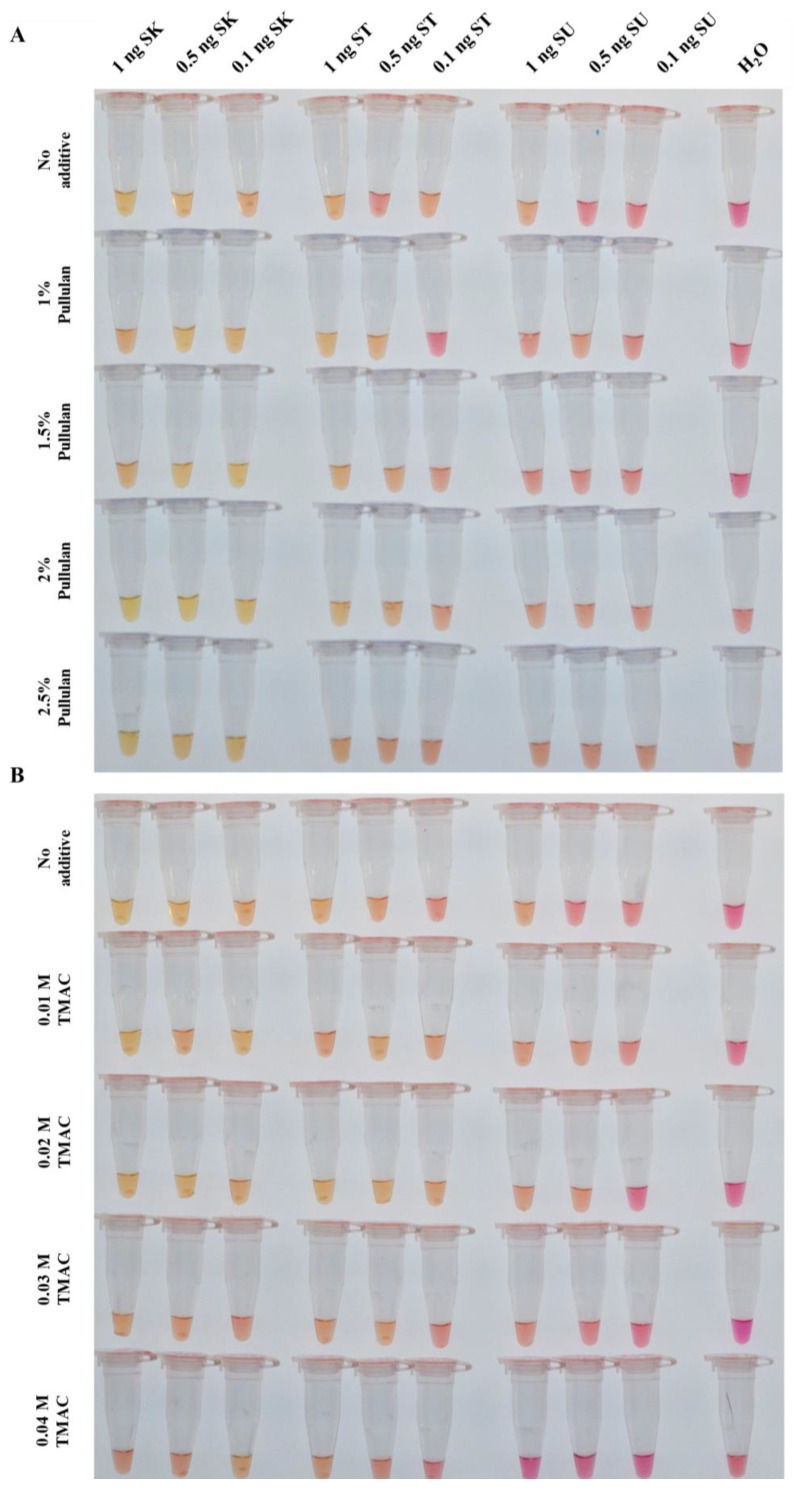

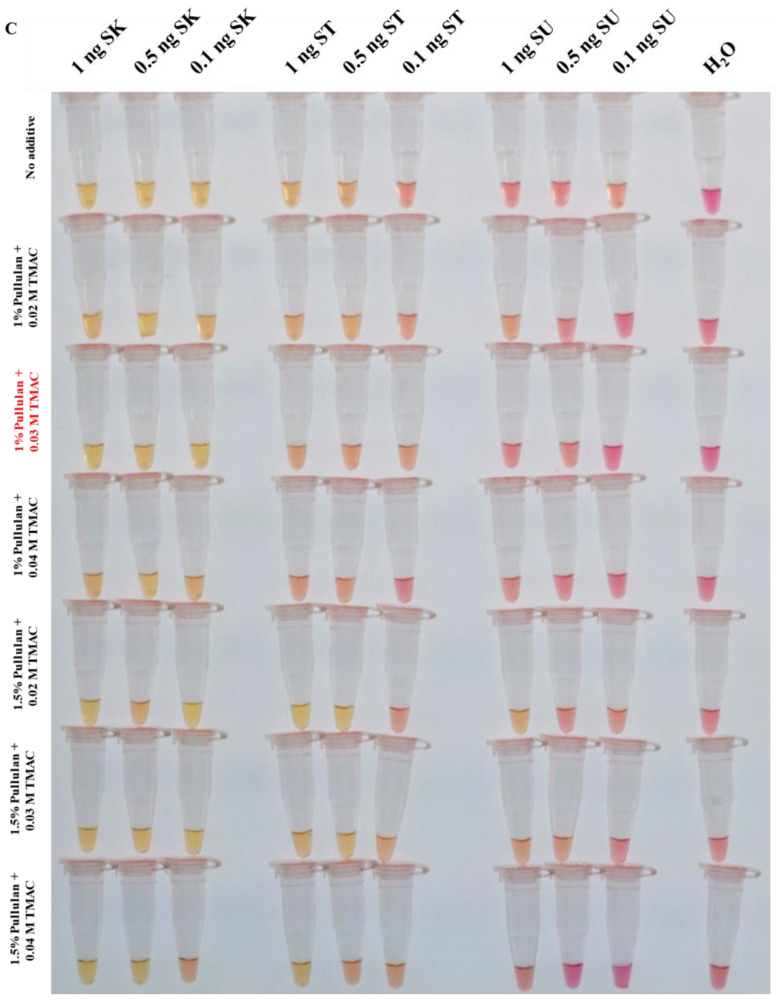
*Sarocladium kiliense*-specific colorimetric loop-mediated isothermal amplification assay (SK-LAMP) with pullulan and tetramethylammonium chloride (TMAC). Different amounts of additives were added into the SK-LAMP reaction mixtures, and DNA templates from *S. kiliense* (SK), *S. strictum* (ST) and *S. summerbellii* (SU) at 0.1, 0.5 and 1 ng were included for amplification. (**A**) SK-LAMP assay supplemented with different concentrations of pullulan (1%, 1.5%, 2% and 2.5%; *v*/*v*). (**B**) SK-LAMP assay supplemented with different concentrations of TMAC (0.01 M, 0.02 M, 0.03 M and 0.04 M). (**C**) SK-LAMP assay supplemented with a combination of pullulan and TMAC at different concentrations: 1% pullulan + 0.02 M TMAC, 1% pullulan + 0.03 M TMAC, 1% pullulan + 0.04 M TMAC, 1.5% pullulan + 0.02 M TMAC, 1.5% pullulan + 0.03 M TMAC and 1.5% pullulan + 0.04 M TMAC. Amongst all the conditions, the combination of 1% pullulan + 0.03 M TMAC was found to be optimal (highlighted in red font).

We tested pullulan at concentrations of 1%, 1.5%, 2% and 2.5% (*v*/*v*) and TMAC at 0.01 M, 0.02 M, 0.03 M and 0.04 M independently. Although non-specific amplification was suppressed, these concentrations were either insufficient to reduce false-positive signals from *S. strictum* DNA or reduced the sensitivity for *S. kiliense* DNA (Figure 2A,B). Overall, lower concentrations at 1% and 1.5% pullulan maintained sensitivity for *S. kiliense* DNA but resulted in false-positives with *S. strictum* DNA, whereas the effects of 0.02 M, 0.03 M and 0.04 M TMAC on the LAMP assay were similar regarding non-specific amplification. To overcome this, we further improved the assay by exploring the combined use of the two additives.

Given the mild effect of individual additives in reducing false-positive signals and the issue of sensitivity reduction at certain concentrations, we further improved the assay by exploring the combined use of the two additives. We evaluated the combination of 1% or 1.5% pullulan with 0.02 M, 0.03 M or 0.04 M TMAC. The combination of 1% pullulan and 0.03 M TMAC significantly inhibited false-positives from *S. strictum* DNA and maintained sensitivity in detecting *S. kiliense* DNA (Figure 2C) across multiple attempts (*n* = 5).

### 3.3. Analytical Specificity and Analytical Sensitivity of SK-LAMP

**Figure 3:**
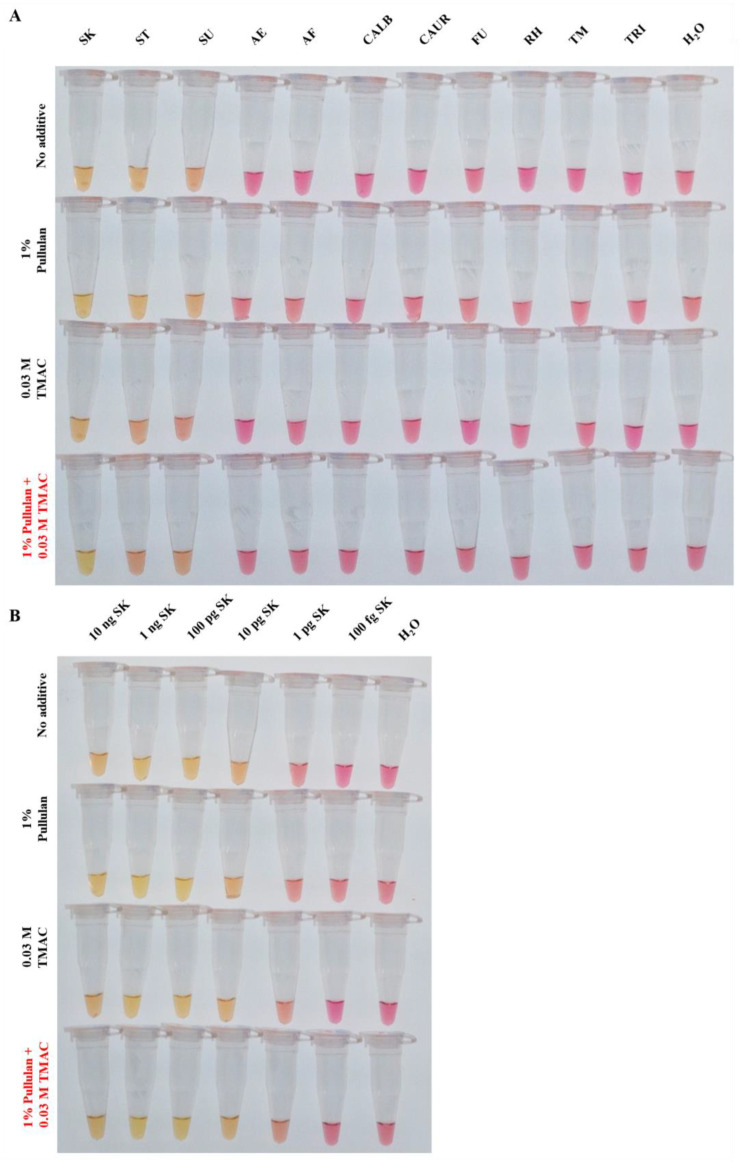
Analytical specificity and analytical sensitivity tests for the optimised *Sarocladium kiliense*-specific colorimetric loop-mediated isothermal amplification assay (SK-LAMP). No additive, 1% pullulan, 0.03 M TMAC or 1% pullulan + 0.03 M TMAC was added in the SK-LAMP reaction mix for (**A**) analytical sensitivity test and (**B**) analytical specificity test. DNA from the fungal species *S. kiliense* (SK), *S. strictum* (ST)*, S. summerbellii* (SU), *Acremonium egyptiacum* (AE), *Aspergillus fumigatus* (AF), *Candida albicans* (CALB), *Candidozyma auris* (CAUR), *Fusarium keratoplasticum* (FU), *Rhizopus microsporus* var. *chinensis* (RH), *Talaromyces marneffei* (TM) and *Trichophyton mentagrophytes* (TRI) were used in the tests.

The analytical specificity of the optimised SK-LAMP assay, using 1% pullulan and 0.03 M TMAC as additives, was tested at an incubation temperature of 68 °C for 45 min. The results showed that the optimised SK-LAMP assay demonstrated no false-positive signals across DNA samples (at 1 ng) from eleven different fungal species (Figure 3A). In the analytical sensitivity test, the optimised SK-LAMP assay demonstrated a limit of detection at 10 pg compared with the no-additive group (Figure 3B). These underscore the potential utility of SK-LAMP for the accurate detection of *S. kiliense* from samples with a diverse fungal background with a high level of analytical specificity and analytical sensitivity.

## 4. Discussion

The identification of *S. kiliense* has traditionally relied on morphological findings and, more recently, molecular testing, such as DNA sequencing [1,6,13,47,48]. For morphological identification, the phenotypic similarity between *S. kiliense* and other filamentous fungi necessitates molecular diagnostics for higher accuracy [11]. Meanwhile, microscopic examination using lactophenol cotton blue-stained slides is challenging because the fungal structures often cannot be well preserved during transfer from the culture plate onto the slides (Figures S2–S5). Although slide culture offers better structural examination, it is tedious and requires an additional 7–10 days, making it impractical for clinical settings. For DNA sequencing, ITS amplification and sequencing are less time-consuming but may not be sustainable in clinical laboratories due to high costs and equipment requirements, especially in developing countries. Nucleic acid amplification tests have also emerged lately; and amongst these, isothermal amplification assays, such as LAMP, RCA, RPA, SDA, HAD and NASBA, are gaining popularity, since the performance of these assays does not require expensive thermocyclers. LAMP assays could also provide further advantages in that they do not require additional instruments for result interpretation; as the results of LAMP assays, when performed in a colorimetric manner, can be read directly by the naked eye. This makes LAMP assays a lot cheaper compared with the other isothermal amplification assays. Moreover, most other isothermal amplification methods, except HDA, require a nucleic acid amplification step lasting more than 1 h, while this can be achieved in around 30–45 min in LAMP assays [16,32,34,49,50]. Since LAMP is a mature isothermal nucleic acid amplification technique, which is widely adopted, suitable for clinical use and is approved for clinical use by the WHO [31,32,33,34,51], its potential for detecting *S. kiliense* was explored in this study. Here, we developed a colorimetric LAMP assay, SK-LAMP, for the detection of *S. kiliense*. Our additional optimisation of the assay overcame the frequent occurrence of false-positive results observed in LAMP assays, providing a more reliable and robust, specific *S. kiliense* detection method than traditional ITS PCR and aiding in the selection of empirical antifungal treatments. Furthermore, with a growing immunodeficient population [52,53], this SK-LAMP assay would enable rapid identification of *S. kiliense* infections, potentially preventing progression to severe invasive infection with poor prognosis.

**Figure 4:**
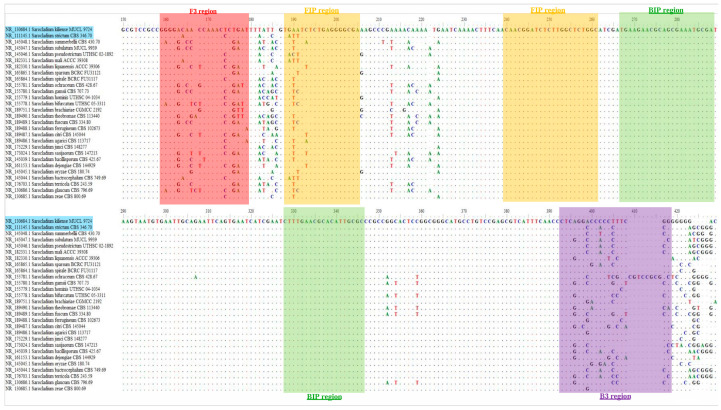
Multiple alignment of the internal transcribed spacer region (ITS) sequences from 28 *Sarocladium* species retrieved from the RefSeq database (Table S1). Regions targeted by the loop-mediated isothermal amplification (LAMP) primer sets used in the *S. kiliense*-specific colorimetric LAMP assay (SK-LAMP) are highlighted in different colours: red—F3 primer (positions 159–179), yellow—FIP primer (positions 188–205 and 240–261), green—BIP primer (positions 266–288 and 328–346) and purple—B3 primer (positions 393–418). *S. kiliense* and *S. strictum* are highlighted in blue.

While the initial prototype without additives detected *S. kiliense* genomic DNA, it also produced false-positive signals with *S. strictum* DNA. This issue likely stemmed from the highly similar ITS regions shared amongst *Sarocladium* species, leading to potential non-specific binding of primers to DNA templates. In particular, *S. strictum* only displays two and four base differences from *S. kiliense* in the ITS target regions of the F3 and B3 primers, respectively (Figure 4). This high sequence similarity may have contributed to the non-specific binding. This highlights the challenge of designing specific primers for differentiating closely related species. To address this issue, we introduced specificity-enhancing additives to our initial prototype assay.

Various chemicals have been reported to inhibit false-positive results in PCR and LAMP reactions (Table 1). In this study, the use of formamide and tween 20 was not tested, as they are not commonly used as additives in LAMP assays. Since our prototype assay was prone to non-specificity, the use of guanidine hydrochloride or bovine serum albumin was also not considered, as these two chemicals are mainly adopted to help enhance sensitivity instead. Moreover, betaine and dimethyl sulphoxide were also not tested, as they have been reported to interfere with LAMP assays [41,54]. As for graphene oxide, our preliminary trial showed that its performance (using 20 ng) was poor because an inhibition of amplification was observed. Pullulan, a strictly linear polysaccharide polymer also known as α-1,4-; α-1,6-glucan, is commonly used in gene delivery, targeted drug therapy, tissue engineering and wound healing [38]. Gao et al. demonstrated that pullulan reduces false-positive signals in LAMP assays by stabilising the primers and reducing the formation of primer dimers in the absence of a target [39]. In PCR, pullulan acts as an enhancer by increasing DNA melting temperature through preferential binding to A/T base pairs, enhancing their stability to approximately that of G/C base pairs [37]. The use of TMAC to inhibit non-specific amplification was first explored in PCR for the AT-rich macronuclear genome of *Paramecium primaurelia* [40]. It has since been used to enhance efficiency and specificity in an isothermal exponential amplification reaction (EXPAR) [55], including a recent reverse transcription–LAMP (RT–LAMP) for SARS-CoV-2 [50]. In this study, we remarkably demonstrated for the first time the potential of combining two additives, 1% pullulan and 0.03 M TMAC, in a LAMP assay to distinguish highly similar LAMP primer binding sites. This approach enlightens further research into the use of different chemical combinations for improving detection efficiency and specificity in LAMP assays.

## 5. Conclusions

This study presents the development of a colorimetric LAMP assay, SK-LAMP, targeting the specific detection of *S. kiliense*. In this SK-LAMP assay, with the presence of 1% (*v*/*v*) pullulan and 0.03 M TMAC, the specificity of detecting *S. kiliense* is improved, preventing the amplification of other *Sarocladium* species, such as *S. strictum*. This allows our assay to detect *S. kiliense* specifically at an amount of as little as 10 pg of fungal DNA in only 45 min. Although our SK-LAMP assay now specifically detects *S. kiliense* DNA, the actual use of SK-LAMP, especially on environmental samples, such as water and soil, may encounter challenges from nucleic acid amplification inhibitors, which are commonly present in the natural environment [51]. Further testing and optimisation may be required to relieve our SK-LAMP assay from these inhibitors [66]. Future studies on *Sarocladium*-related diseases could benefit from the development of this SK-LAMP assay. Many case reports currently identify fungal species only at the genus level and often rely on morphological findings to identify *Acremonium* and *Sarocladium* species, which is unreliable for closely related species. A rapid SK-LAMP assay would enable quicker and more accurate identification of *S. kiliense*.

## Figures and Tables

**Table 1:** Different chemical additives commonly used in loop-mediated isothermal amplification (LAMP) assays.

Additive	Functions in LAMP Assays	References
Betaine	· Denatures secondary structure, preventing secondary structure formation in GC-rich region · Increases specificity but decreases sensitivity · Increases DNA accessibility	[56,57,58,59,60,61,62,63,64]
Bovine serum albumin	· Increases efficiency and sensitivity · Reduces gene-amplification inhibitory effect caused by melanin, humic acid and urea · Reduces potential DNA/RNA mismatch or relieves interference during gene amplification	[41,65,66,67]
Dimethyl sulfoxide	· Inhibits non-specific amplification but also the activity of *Bst* 2.0 WarmStart DNA polymerase · Prevents formation of secondary structures in DNA templates or primers	[41,63,64,68]
Formamide	· Stabilises double helix by binding to minor or major grooves of DNA template	[41]
Graphene oxide	· Adsorbs primers and DNA template to inhibit primer hybridisation · Enhances specificity	[69,70,71]
Guanidine hydrochloride	· Increases sensitivity · Denatures nucleic acid and prevents formation of secondary and tertiary structures · Disrupts hydrogen bonds between water molecules and nucleic acids, thus increasing solubility of hydrophobic surfaces of the DNA and facilitating denaturation of DNA from double strand into single strand	[64,72,73,74,75]
Pullulan	· Stabilises multiple primers to reduce dimer formation · Acts as an enhancer by increasing DNA melting temperature through preferential binding to A/T base pairs, thus enhancing their stability to approximately that of G/C base pairs	[37,38,39,64,72,76]
Tetramethylammonium chloride (TMAC)	· Increases efficiency and specificity · Reduces potential DNA/RNA mismatch or relieves interference during gene amplification	[40,41,55,64,72]
Tween 20	· Increases sensitivity but also non-specific amplification	[41]

## Data Availability

The original contributions presented in the study are included in the article/Supplementary Material, further inquiries can be directed to the corresponding authors.

## Supplementary Materials

The following supporting information can be downloaded at: https://www.mdpi.com/article/10.3390/jof10120857/s1, Table S1. *Sarocladium* species included for multiple alignment based on internal transcribed spacer region (ITS) sequences. Figure S1. Results for the prototype colorimetric loop-mediated isothermal amplification (LAMP) assay performed at 68 °C. The assay was tested with *Sarocladium kiliense*, *S. strictum* and *S. summerbellii* DNA at 1, 0.5 and 0.1 ng. Water was used as a negative control. Figure S2. Macro- and micromorphologies of a 14-day culture of *Sarocladium kiliense* incubated at 25 ± 1 °C on Sabouraud dextrose agar (SDA) and potato dextrose agar (PDA). a: Obverse colony morphology on SDA; b: Reverse colony morphology on SDA; c: Detached ellipsoidal and cylindrical conidia near slender hyphae from the colony on SDA under a 100× light microscope; d: Obverse colony morphology on PDA; e: Reverse colony morphology on PDA; f: Detached ellipsoidal and cylindrical conidia near an entangled ball of hyphae from the colony on PDA under a 100× light microscope. All the photographs were taken with an iPhone 15 (Apple Inc., Cupertino, CA, USA) using the original camera application. Figure S3. Macro- and micromorphologies of a 14-day culture of *Sarocladium strictum* incubated at 25 ± 1 °C on Sabouraud dextrose agar (SDA) and potato dextrose agar (PDA). a: Obverse colony morphology on SDA; b: Reverse colony morphology on SDA; c: Detached ellipsoidal and cylindrical conidia near slender hyphae from the colony on SDA under a 100× light microscope; d: Obverse colony morphology on PDA; e: Reverse colony morphology on PDA; f: Detached ellipsoidal and cylindrical conidia near an entangled ball of hyphae from the colony on PDA under a 100× light microscope. All the photographs were taken with an iPhone 15 (Apple Inc., Cupertino, CA, USA) using the original camera application. Figure S4. Macro- and micromorphologies of a 14-day culture of *Sarocladium summerbellii* incubated at 25 ± 1 °C on Sabouraud dextrose agar (SDA) and potato dextrose agar (PDA). a: Obverse colony morphology on SDA; b: Reverse colony morphology on SDA; c: Detached ellipsoidal and cylindrical conidia near slender hyphae from the colony on SDA under a 100× light microscope; d: Obverse colony morphology on PDA; e: Reverse colony morphology on PDA; f: Detached ellipsoidal and cylindrical conidia near an entangled ball of hyphae from the colony on PDA under a 100× light microscope. All the photographs were taken with an iPhone 15 (Apple Inc., Cupertino, CA, USA) using the original camera application. Figure S5. Macro- and micromorphologies of a 14-day culture of *Acremonium egyptiacum* incubated at 25 ± 1 °C on Sabouraud dextrose agar (SDA) and potato dextrose agar (PDA). a: Obverse colony morphology on SDA; b: Reverse colony morphology on SDA; c: Detached ellipsoidal and cylindrical conidia near slender hyphae from the colony on SDA under a 100× light microscope; d: Obverse colony morphology on PDA; e: Reverse colony morphology on PDA; f: Detached ellipsoidal and cylindrical conidia near an entangled ball of hyphae from the colony on PDA under a 100× light microscope. All the photographs were taken with an iPhone 15 (Apple Inc., Cupertino, CA, USA) using the original camera application.
