# Supplementary material for: Enhanced Specificity in Colorimetric LAMP Assay for Sarocladium kiliense Detection Using a Combination of Two Additives
Source: J Fungi (Basel). 2024 Dec 11;10(12):857. doi: 10.3390/jof10120857 (PMC11678401; doi:10.3390/jof10120857)
Supplement: Supplementary file 1 [file jof-10-00857-s001.zip › SK-LAMP - Supplementary Figures Legends & Supplementary Figures.pdf]

Table S1. *Sarocladium* species included for multiple alignment based on internal transcribed spacer region (ITS) sequences.

Figure S1. Results for the prototype colorimetric loop-mediated isothermal amplification (LAMP) assay performed at 68°C. The assay was tested with *Sarocladium kiliense*, *S. strictum* and *S. summerbellii* DNA at 1, 0.5 and 0.1 ng. Water was used as a negative control.

Figure S2. Macro- and micromorphologies of a 14-day culture of *Sarocladium kiliense* incubated at 25 ± 1 °C on Sabouraud dextrose agar (SDA) and potato dextrose agar (PDA). a: Obverse colony morphology on SDA; b: Reverse colony morphology on SDA; c: Detached ellipsoidal and cylindrical conidia near slender hyphae from the colony on SDA under a 100× light microscope; d: Obverse colony morphology on PDA; e: Reverse colony morphology on PDA; f: Detached ellipsoidal and cylindrical conidia near an entangled ball of hyphae from the colony on PDA under 100× light microscope. All the photos were taken with an iPhone 15 (Apple Inc., Cupertino, CA, USA) using the original camera application.

Figure S3. Macro- and micromorphologies of a 14-day culture of *Sarocladium strictum* incubated at 25 ± 1 °C on Sabouraud dextrose agar (SDA) and potato dextrose agar (PDA). a: Obverse colony morphology on SDA; b: Reverse colony morphology on SDA; c: Detached ellipsoidal and cylindrical conidia near slender hyphae from the colony on SDA under a 100× light microscope; d: Obverse colony morphology on PDA; e: Reverse colony morphology on PDA; f: Detached ellipsoidal and cylindrical conidia near an entangled ball of hyphae from the colony on PDA under 100× light microscope. All the photos were taken with an iPhone 15 (Apple Inc., Cupertino, CA, USA) using the original camera application.

Figure S4. Macro- and micromorphologies of a 14-day culture of *Sarocladium summerbellii* incubated at 25 ± 1 °C on Sabouraud dextrose agar (SDA) and potato dextrose agar (PDA). a: Obverse colony morphology on SDA; b: Reverse colony morphology on SDA; c: Detached ellipsoidal and cylindrical conidia near slender hyphae from the colony on SDA under a 100× light microscope; d: Obverse colony morphology on PDA; e: Reverse colony morphology on PDA; f: Detached ellipsoidal and cylindrical conidia near an entangled ball of hyphae from the colony on PDA under 100× light microscope. All the photos were taken with an iPhone 15 (Apple Inc., Cupertino, CA, USA) using the original camera application.

Figure S5. Macro- and micromorphologies of a 14-day culture of *Acremonium egyptiacum* incubated at 25 ± 1 °C on Sabouraud dextrose agar (SDA) and potato dextrose agar (PDA). a: Obverse colony morphology on SDA; b: Reverse colony morphology on SDA; c: Detached ellipsoidal and cylindrical conidia near slender hyphae from the colony on SDA under a 100× light microscope; d: Obverse colony morphology on PDA; e: Reverse colony morphology on PDA; f: Detached ellipsoidal and cylindrical conidia near an entangled ball of hyphae from the colony on PDA under 100× light microscope. All the photos were taken with an iPhone 15 (Apple Inc., Cupertino, CA, USA) using the original camera application.

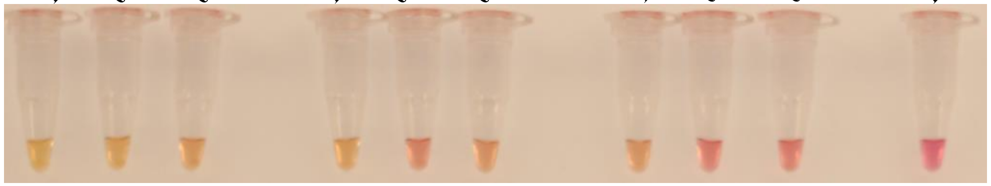
1 ng SK
0.5 ng SK
0.1 ng SK
1 ng ST
0.5 ng ST
0.1 ng ST
1 ng SU
0.5 ng SU
0.1 ng SU
H2O
No additive

Figure S1

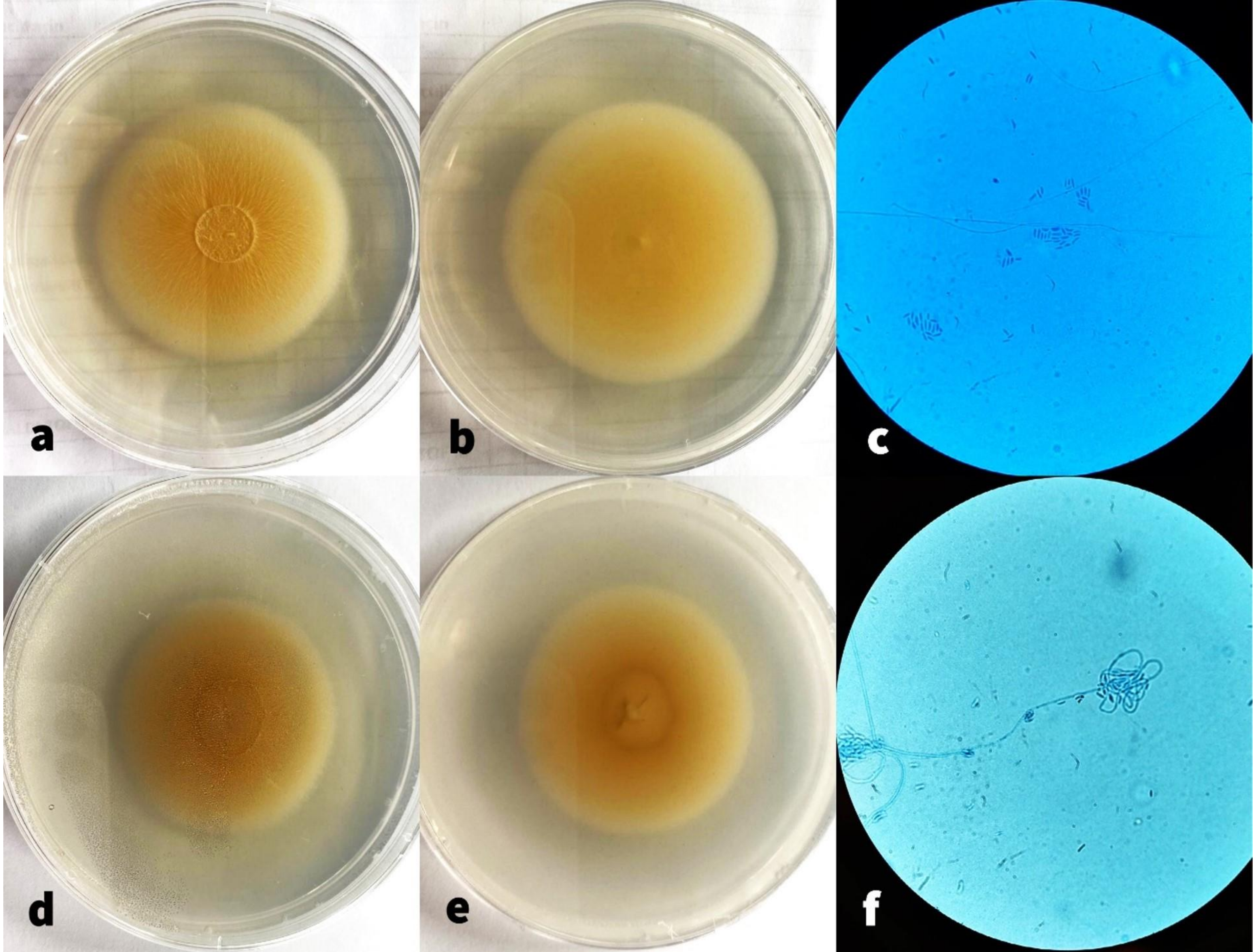

Figure S2

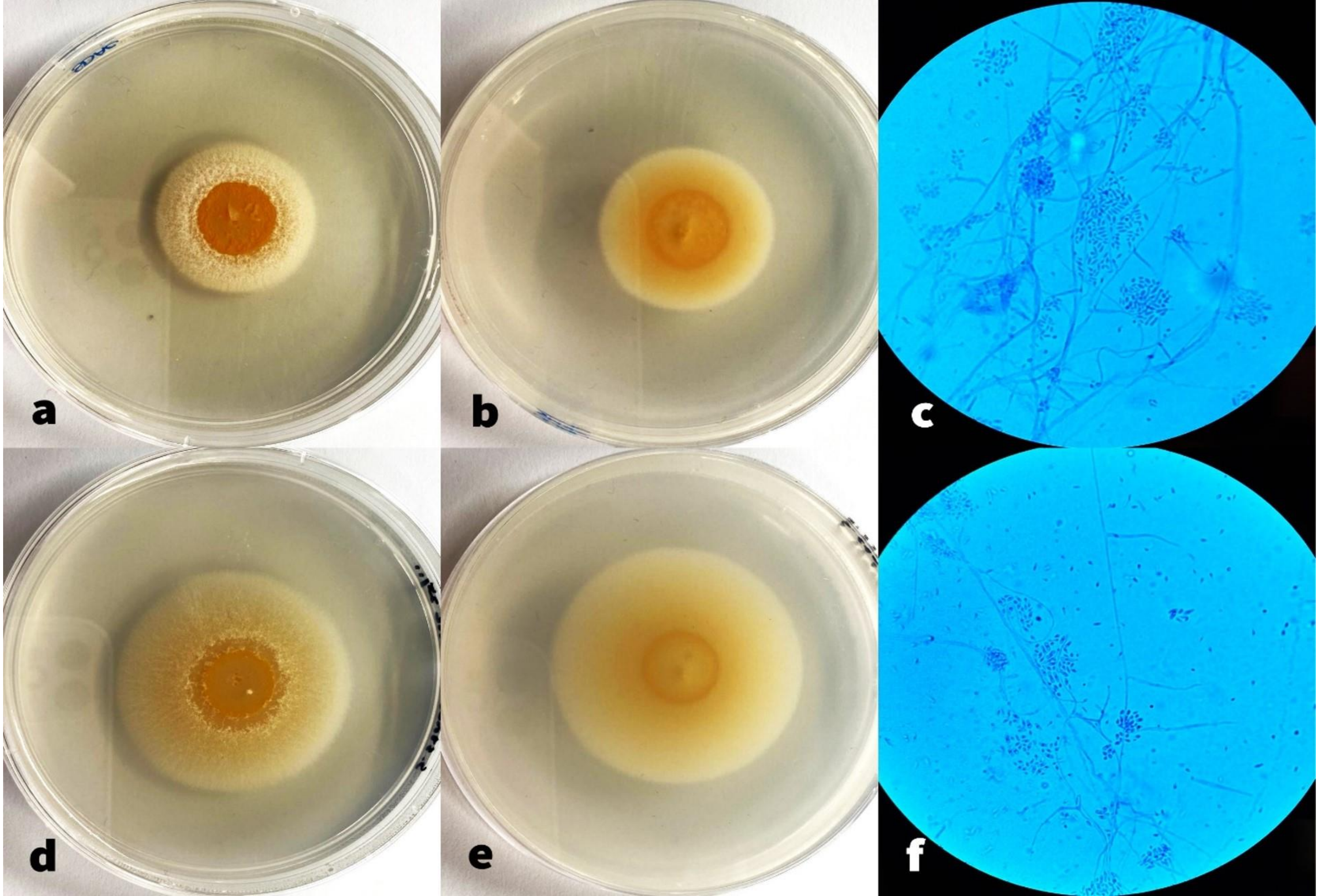

Figure S3

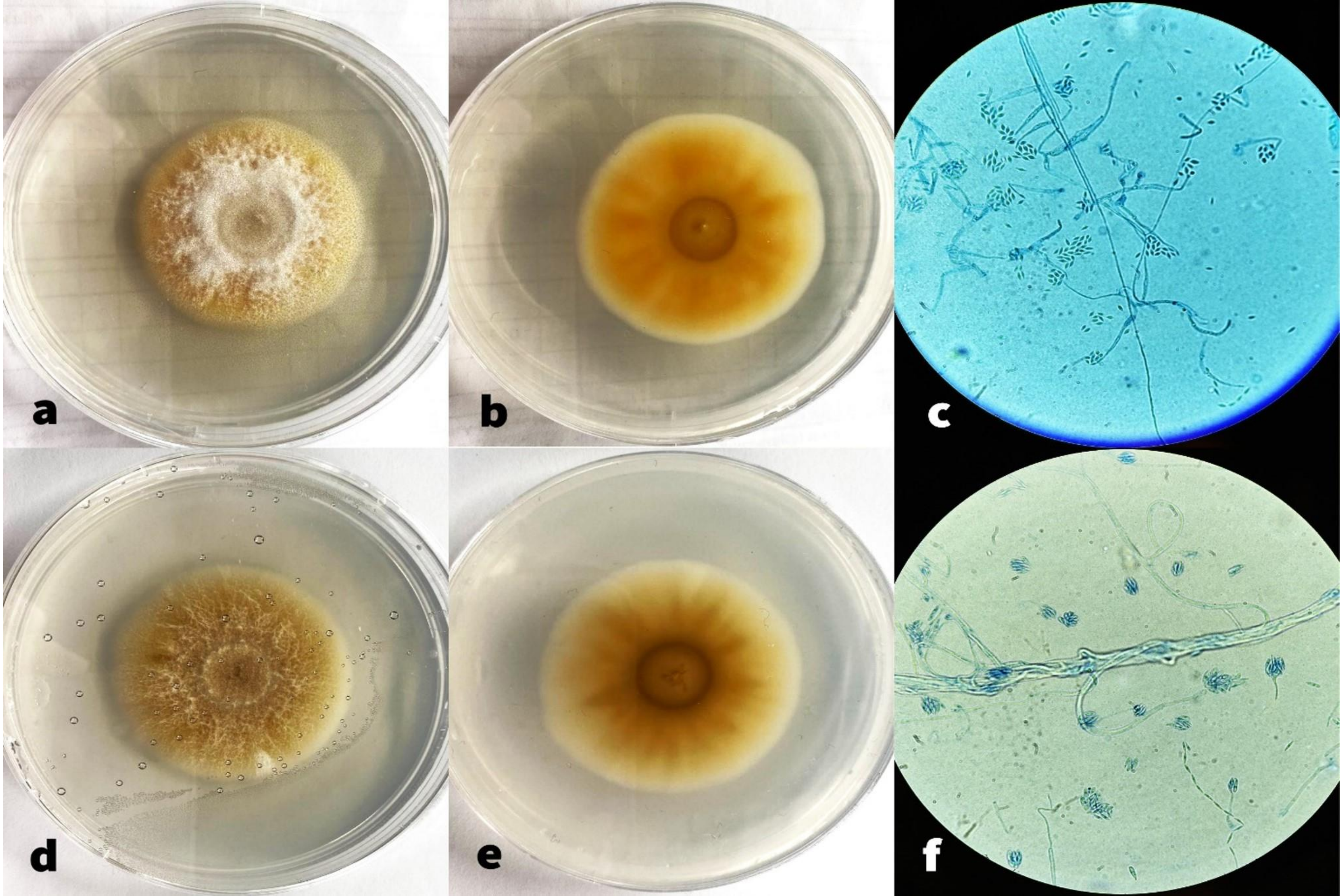

Figure S4

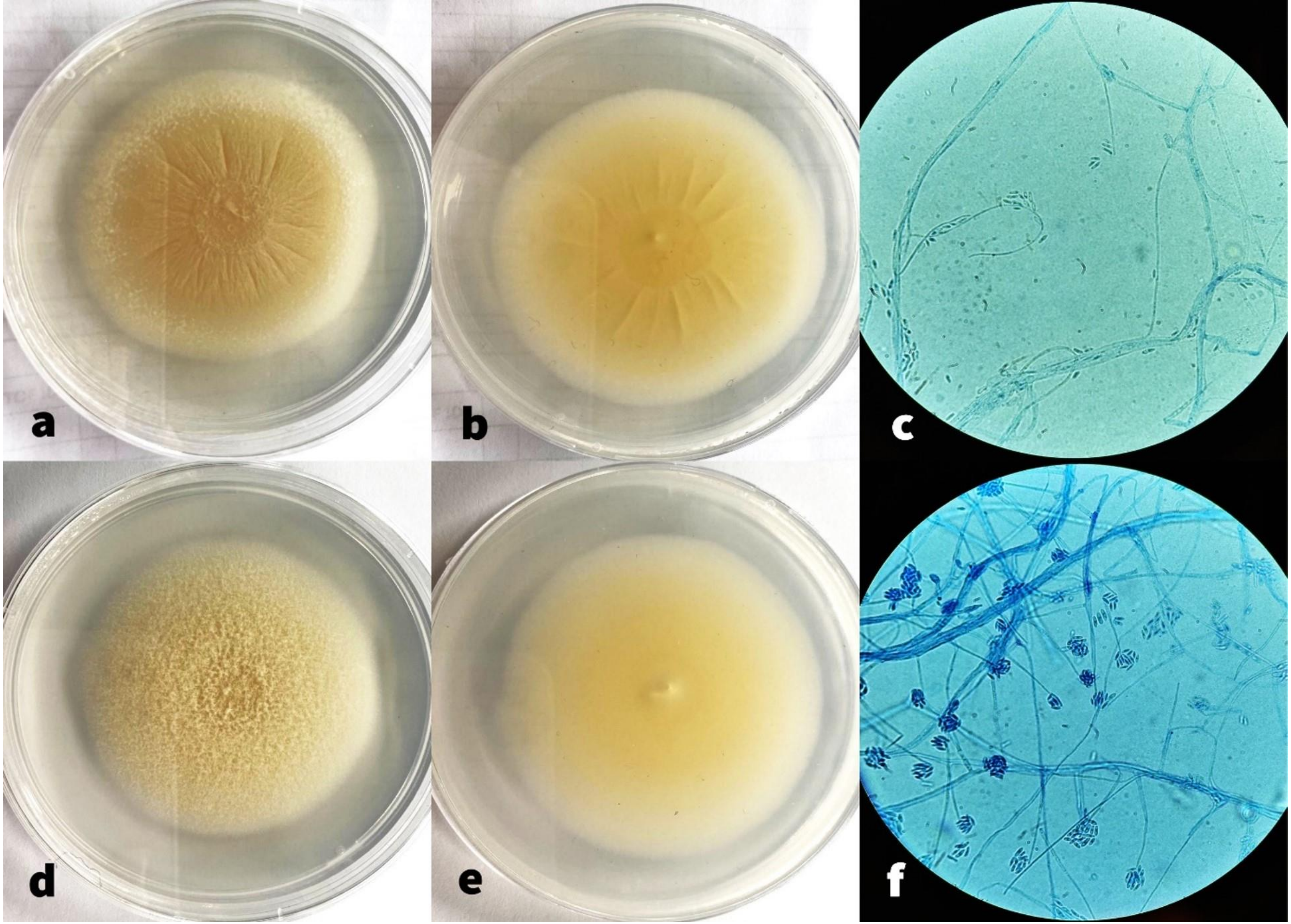

Figure S5
