# Supplementary material for: Enhanced Specificity in Colorimetric LAMP Assay for Sarocladium kiliense Detection Using a Combination of Two Additives
Source: J Fungi (Basel). 2024 Dec 11;10(12):857. doi: 10.3390/jof10120857 (PMC11678401; doi:10.3390/jof10120857)
Supplement: Supplementary file 1 [file jof-10-00857-s001.zip › SK-LAMP - Supplementary table.pdf]

Table S1. *Sarocladium* species included for multiple alignment based on internal transcribed spacer region (ITS) sequences.

Species	Strain	Nucleotide accession number
Sarocladium agarici	CBS 113717	NR_189486.1
Sarocladium bacillisporum	CBS 425.67	NR_145039.1
Sarocladium bactrocephalum	CBS 749.69	NR_145044.1
Sarocladium bifurcatum	UTHSC 05-3311	NR_155778.1
Sarocladium brachiariae	CGMCC 2192	NR_189751.1
Sarocladium citri	CBS 145044	NR_189487.1
Sarocladium dejongiae	CBS 144929	NR_161153.1
Sarocladium ferrugineum	CBS 102673	NR_189488.1
Sarocladium fuscum	CBS 334.80	NR_189489.1
Sarocladium gamsii	CBS 707.73	NR_155780.1
Sarocladium glaucum	CBS 796.69	NR_130686.1
Sarocladium hominis	UTHSC 04-1034	NR_155779.1
Sarocladium junci	CBS 148277	NR_175229.1
Sarocladium kiliense	MUCL 9724	NR_130684.1
Sarocladium liquanensis	ACCC 39306	NR_182330.1
Sarocladium mali	ACCC 39308	NR_182331.1
Sarocladium ochraceum	CBS 428.67	NR_155781.1
Sarocladium oryzae	CBS 180.74	NR_145045.1
Sarocladium pseudostrictum	UTHSC 02-1892	NR_145046.1
Sarocladium sasijaorum	CBS 147213	NR_173024.1
Sarocladium sparsum	BCRC FU31121	NR_165865.1
Sarocladium spirale	BCRC FU31117	NR_165864.1
Sarocladium strictum	CBS 346.70	NR_111145.1
Sarocladium subulatum	MUCL 9939	NR_145047.1
Sarocladium summerbellii	CBS 430.70	NR_145048.1
Sarocladium terricola	CBS 243.59	NR_176703.1
Sarocladium theobromae	CBS 113440	NR_189490.1
Sarocladium zeae	CBS 800.69	NR_130685.1
